# Causes of maternal mortality in Sub-Saharan Africa: A systematic review of studies published from 2015 to 2020

**DOI:** 10.7189/jogh.11.04048

**Published:** 2021-10-09

**Authors:** Reuben Musarandega, Michael Nyakura, Rhoderick Machekano, Robert Pattinson, Stephen Peter Munjanja

**Affiliations:** 1 School of Health Systems and Public Health, University of Pretoria, South Africa; 2 Department of Obstetrics and Gynaecology, Victoria Falls Hospital, Zimbabwe; 3 Biostatistics and Epidemiology Department, Faculty of Medicine and Health Sciences, Stellenbosch University, Cape Town, South Africa; 4 Research Centre for Maternal, Fetal, Newborn & Child Health Care Strategies, University of Pretoria, South Africa; 5 Unit of Obstetrics and Gynaecology, Faculty of Medical and Health Sciences, University of Zimbabwe, Harare, Zimbabwe

## Abstract

**Background:**

Maternal deaths remain high in Sub-Saharan Africa (SSA) and their causes of maternal death must be analysed frequently in this region to guide interventions.

**Methods:**

We conducted a systematic review of studies published from 2015 to 2020 that reported the causes of maternal deaths in 57 SSA countries. The objective was to identify the leading causes of maternal deaths using the international classification of disease – 10^th^ revision, for maternal mortality (ICD-MM). We searched PubMed, WorldCat Discovery Libraries Worldwide (including Medline, Web of Science, LISTA and CNHAL databases), and Google Scholar databases and citations, using the search words “maternal mortality”, “maternal death”, “pregnancy-related death”, “reproductive age mortality” and “causes” as MeSH terms or keywords. The last date of search from all databases was 21 May 2021. We included original research articles published in English and excluded articles that mentioned SSA country names without study results for those countries, studies that reported death from a single cause or assigned causes of death using computer models or incompletely broke down the causes of death. We exported, de-duplicated and screened the searches electronically in EndNote version 20. We selected the final articles by reading the titles, abstracts and full texts. Two authors searched the articles and assessed the risk of bias using a tool adapted from Montoya and others. Data from the articles were extracted onto an Excel worksheet and the deaths classified into ICD-MM groups. Proportions were calculated with 95% confidence intervals and compared for deaths attributed to each cause and ICD-MM group. We compared the results with WHO and Global Burden of Disease (GDB) estimates.

**Results:**

We identified 38 studies that reported 11 427 maternal and four incidental deaths. Twenty-one of the third-eight studies were retrospective record reviews. The leading causes of death (proportions and 95% confidence intervals (CI)) were obstetric hemorrhage: 28.8% (95% CI = 26.5%-31.2%), hypertensive disorders in pregnancy: 22.1% (95% CI = 19.9%-24.2%), non-obstetric complications: 18.8% (95% CI = 16.4%-21.2%) and pregnancy-related infections: 11.5% (95% CI = 9.8%-13.2%). The studies reported few deaths of unknown/undetermined and incidental causes.

**Conclusions:**

Limitations of this review were the failure to access more data from government reports, but the study results compared well with WHO and GDB estimates. Obstetric hemorrhage, hypertensive disorders in pregnancy, non-obstetric complications, and pregnancy-related infections are the leading causes of maternal deaths in SSA. However, deaths from incidental causes are likely under-reported in this region. SSA countries must continue to invest in health information systems that collect and publishes comprehensive, quality, maternal death causes data. A publicly accessible repository of data sets and government reports for causes of maternal death will be helpful in future reviews. This review received no specific funding and was not registered.

Knowledge of the leading causes of maternal deaths in different regions and countries is imperative. It helps governments, global health organisations, and local partners to prioritise interventions to reduce maternal mortality. However, some global health regions lack empirical data on the causes of maternal mortality [1]. Sub-Saharan Africa (SSA), the region with the highest maternal mortality, lacks these data [2-4].

In 2017, the Maternal Mortality Estimation Interagency Group (MMEIG) estimated that 295 000 maternal deaths occurred globally, of which 196 000 (66%) were from SSA [2]. SSA maternal mortality ratio (MMR) was 542 deaths per 100 000 live births compared to a global ratio of 216 deaths per 100 000 live births. Maternal mortality interventions must be prioritised in SSA countries to reduce the MMR from these high levels and meet the Sustainable Development Goals (SDG). SDG 3.1 target is for individual countries to reduce their MMRs by two-thirds from their 2010 baseline. Countries with MMR above 420 have to reduce their MMRs to no greater than 140 maternal deaths per 100 000 live births [5,6]. Cause-specific data are needed to prioritise interventions in this context.

Systematic reviews and meta-analyses often address the non-availability of comprehensive data in specific subject areas. We did not find recent systematic reviews of the causes of maternal deaths in SSA. A 2006 global review included just seven SSA studies [7]. A 2014 global analysis did not categorise the maternal deaths into the groups recommended by the International Classification of Diseases 10th Revision for Maternal Mortality (ICD-MM) [8]. Despite contributing two-thirds of global maternal deaths, the SSA region provided only 29% of the deaths analysed in this study. Other more recent reviews were more restricted in their scope. They reviewed the causes of institutional maternal deaths [9], causes of maternal mortality in adolescent mothers [10], maternal mortality levels [11], HIV-attributed maternal mortality [12], case fatality rates for severe maternal morbidity [13], and maternal near-miss [14].

This systematic review searched for studies published from 2015 to 2020 reporting causes of maternal deaths in SSA. It’s objective was to identify the leading causes of maternal deaths in the SSA region using ICD-MM groups. The start year, 2015, was selected to narrow the search to studies that used ICD-MM in identifying the causes of death. The rationale was that there was no recent review of published articles to identify the leading causes of maternal mortality in the SSA region. The review provides a benchmark for future empirical studies that identifies the leading causes of maternal mortality in SSA countries. SSA needs such studies to accelerate the reduction of maternal mortality towards the SDG 3.1 target.

## MATERIALS AND METHODS

### Search criteria

We followed the Preferred Reporting Items for Systematic Reviews and Meta-Analysis (PRISMA) 2020 guidelines in conducting this systematic review. Guided by the stated objective, we built search strategies using the words “maternal mortality”, “reproductive age mortality”, “pregnancy-related deaths”, “causes” and SSA country names. We searched PubMed, WorldCat Discovery Libraries Worldwide (including Medline, Web of Science, LISTA and CNHAL databases), and Google Scholar (last searched 21/05/2021) for studies that reported causes of maternal deaths in SSA, published from 2015 to 2020. We used “*include related terms”* options in the searches and combined the search terms using Boolean operators “OR” and “AND”. We used MeSH terms in PubMed search and Keywords expanded to “related terms” in World Cat Discovery and Google Scholar searches. Additional searches were done using citations and “similar” or “related articles” options for included articles. The search period started from 2015 to limit the review to studies containing data collected from 2010 onwards, which would have used the ICD-MM. We performed the search from 16 December 2020 to 21 March 2021 and repeated it from 13 to 21 May 2021. For the detailed strategy for each search see Table S1 in the **Online Supplementary Document**.

### Inclusion and exclusion criteria

We included original research articles published in English that reported causes of maternal deaths and mentioned SSA countries. We excluded articles without study results for the SSA countries mentioned in them, studies that evaluated interventions, studied a single cause of death, assigned death causes using computer models, incompletely broke down the causes of death (less than 75% of total deaths identified), without full articles or reported data collected before 2010 (before the introduction of ICD-MM) or reported results contained in other selected studies. Unpublished government reports for Zimbabwe and South Africa, which the authors managed to access, were also included. We searched for a central repository of government reports but found none. We could not find additional government reports from the databases’ and open internet searches, and enquiries with relevant UN agencies. Two authors searched for and screened the articles independently, and discussed and agreed on the selected articles. Differences were resolved by the last author, who verified the eligibility of the selected articles and the assigning of the causes of death into ICD-MM groups.

### Screening of the articles

Two authors screened the articles using titles and abstracts, and reading full-text versions when abstracts indicated that the study reported the causes of maternal death. All articles identified from the searches were exported to EndNote version 20, and duplicates removed electronically. The articles were initially screened electronically using keywords “maternal”, “death”, “mortality” and “causes,” combining the search terms using the Boolean Operators “AND” and “OR”. The remaining articles were screened manually using study titles and abstracts. The verification of eligibility of the selected articles was done using the study titles and abstracts.

### Data extraction

The first author extracted the data from the included articles onto an adapted Critical Appraisal Skills Programme (CASP) form in Microsoft Excel (Microsoft Inc, Seattle, USA), recording the article’s first author name, year published, study description or title, study implementer (study group or government), the period covered by the data, study design, study setting, source of maternal death data, the definition of maternal death used, whether the study included community deaths, the method used to assign the causes of death, country and SSA region where the study was done, the total number of maternal deaths reported and breakdown of maternal deaths by cause of death – categorised into ICD-MM groups (Table S2 in the **Online Supplementary Document**). The primary study outcome was the number of deaths attributed to each cause of maternal death. The number of deaths for each cause was identified from the results section of the articles and not abstracts. For the PRISMA diagram with the search method and results see Figure S1 in the **Online Supplementary Document**.

### Data processing and synthesis

We synthesised the data in an Excel data extraction worksheet. As data were extracted from each selected article, the different causes of death reported in the studies were added to the data extraction template. In the process, each cause of death was assigned to the relevant cause of death group, using the ICD-MM manual. Each eligible study was recorded on the datasheet with the number of deaths recorded under the applicable cause of death. When the breakdown of causes of death was given in percentages, we calculated the number of deaths using the percentages and total deaths reported in the study. No studies were excluded at the synthesis stage.

### Risk-of-Bias assessment

Standard risk-of-bias assessment tools such as the Risk of Bias in randomised trials (RoB 2 tool), Risk Of Bias in Non-randomised Studies (ROBINS-I tool) and Risk of Bias due to Missing Evidence in synthesis (ROB_ME) were considered but deemed inapplicable for this review. They are designed for systematic review of studies measuring intervention effects. Instead, the risk of bias was assessed using a tool adapted from Montoya and others’ study, which reviewed hospital maternal mortality levels in SSA. This tool assesses different types of bias, depending on the kind of studies reviewed and the biases that affect them [11]. In their review, Montoya and others assessed four types of bias as follows: selection bias from the definition of maternal death used in the studies, information bias from the sources of data, selection bias from the length of follow up of the women, and selection bias from inclusion or exclusion of deaths in early pregnancy. We adapted the tool to four criteria that suited our review. Using the adapted tool, we assessed: information bias from the source of maternal death data used in the studies, missing data bias from the completeness of the cause-of-death data against total deaths reported, selection bias from the use or non-use of ICD-MM in assigning causes of death in the study, measurement bias from competence levels of persons who assigned the causes of death. Under each criterion, the risk of bias was rated and scored as low (1), medium (2), or high (3) (**Table 1**). We assigned the overall risk of bias rating for each study as low (average score less than 1.5), medium (average score ranging 1.5 to less than 2), and low (average score ranging from 2 to 3) (See Table S3 in the **Online Supplementary Document**). Studies with a high risk of bias were to be excluded from analysis.

**Table 1:** Risk of bias assessment criteria

	Definition of the risk of bias criterion			
**Risk-of-bias (rating score)**	**Source of data for maternal death in the study (information bias)**	**Completeness of cause-of-death data in the study (missing data bias)**	**Use of ICD-MM in assigning the causes of death in the study (selection bias)**	**Competence level of persons who assigned the causes of death (measurement bias)**
Low (1)	Health facility records	≥90% deaths assigned causes	Use of ICD-MM definition stated	Expert panels / MDSR committees
Medium (2)	MDSR audits	75-90% deaths assigned causes	Not stated but data collected 2015 onwards	Primary clinical assessments in facility records
High (3)	Community surveys with VAs	<75% deaths assigned causes	Not stated and data collected 2010 to 2014	Not stated
Rationale for ratings	Health facility records contain all details of events leading to the death. MDSR audits contain summaries, and VA reports lack clinical detail.	The rating looks at increasing the risk of bias. WHO recommends ≥75% completeness to rate the data as reasonable for use in review studies.	Mention of ICD-MM use assures that the standard definitions and classification of the causes of death was followed. Data from 2015 gives near assurance, and data before that may not have used ICD-MM classification.	Deaths assessed by an expert or CEMD/MDSR committees are more comprehensive and accurate in their assessments. Facility clinical assessments may be biased or less competent.

VA – verbal autopsy, CEMD/MDSR – confidential enquiry into maternal deaths/maternal death surveillance and response

### Data analysis

We calculated the total number of deaths reported in all the studies, total number of deaths belonging to each ICD-MM group and the total number of deaths reported under each specific cause of death. Proportions of deaths attributed to each ICD-MM group and specific causes for all SSA and sub-regions (East and Central, Southern and West Africa) were calculated. The leading causes of death in SSA and its sub-regions were identified by ranking the proportions of deaths. The leading causes of death from this study were compared with WHO and Global Burden of Disease (GBD) cause-of-death estimates, using the rankings of the causes of death from each study. Meta-analysis was not performed because this review assessed all causes of maternal deaths reported in the studies and not one cause. Heterogeneity and sensitivity analysis was not performed as these apply to meta-analysis. The risk of bias from missing results was assessed using completeness of reporting the causes of death. The robustness of the synthesised results was assessed using 95% confidence intervals for the proportions of deaths.

### Ethics approvals

The systematic review was not registered, but conducted under a study protocol approved by the Medical Research Council of Zimbabwe (MRCZ/A/2613) and the University of Pretoria Faculty of Science Research Ethics Committee (339/2019).

## RESULTS

A total of 6 519 titles and abstracts were identified and screened from PubMed, 8 812 from World Cat Discovery Worldwide Libraries, 245 from Google Scholar and 26 from citation and similar articles searches. From these, 149 full-text articles were evaluated, and 38 studies included for final analysis. The 38 studies reported 11 427 maternal deaths and four (4) incidental deaths from 20 SSA countries (range of maternal deaths reported in the studies: 18 to 2 326). For the details and data for each included study see Table S2 in the **Online Supplementary Document**.

Twenty-three of the studies were retrospective health record reviews [15-37]. The other studies were: case-control (n = 1) [38], Confidential Enquiries into Maternal Deaths (CEMD) or Maternal Death Surveillance and Response (MDSR) audits (n = 4) [39-42], cross-sectional facility and community surveys (n = 4) [43-46], prospective facility and community studies (n = 2) [47,48], Reproductive Age Mortality Study (RAMOS) (n = 2) [49,50], pre and post evaluation (n = 1) [51], and step-wedge randomised controlled trials (RCT) (n = 1) [52]. The methods of assigning the causes of death were: study expert panels (n = 21) [17,18,21,22,24,26,28,29,33-35,37,39,43-46,48-50,52], facility/MDSR audit teams (n = 8) [16,23,25,31,41,42,51,53], routine clinical assessments (n = 5) [19,20,27,30,32], physician verbal autopsy coders (n = 1) [47], and not stated (n = 3) [15,36,38]. South Africa contributed 21% of the deaths from 2 studies [22,41], Nigeria 18% from 13 studies [15,21,23-25,28-31,35,37,46,48], Tanzania 15% from 3 studies [16,18,39], and Zimbabwe 9% from 2 studies [20,42] (See Table S4 in the **Online Supplementary Document**). Risk of bias was rated as low in 20 studies and medium in 14 studies (Table S.2 in the **Online Supplementary Document** ). No studies rated as high in risk of bias; hence, none was excluded for high risk of bias. Only 6 out of the 38 studies reported incomplete cause-of-death data – three with 76%, 81% and 96%, and three with 99% completeness. Thus, the risk of bias for the pooled data was judged as low.

### Leading causes of maternal mortality in SSA

Obstetric haemorrhage was the leading group of maternal mortality causes (proportion of deaths; 95% confidence interval) (28.8%; 95% CI = 26.5%-31.2%) followed by hypertensive disorders in pregnancy (22.1%; 95% CI = 19.9%-24.2%), non-obstetric complications (18.8%; 95% CI = 16.4%-21.2%) and pregnancy-related infections (11.5%; 95% CI = 9.8%-13.2%). Very few deaths (4) were due to coincidental causes (**Table 2**). Postpartum haemorrhage was the leading cause of death in the obstetric haemorrhage group. Pre-eclampsia/eclampsia was the leading cause of hypertensive disorders in pregnancy group, and puerperal sepsis the leading cause in the pregnancy-related infections group (See Table S5 in the **Online Supplementary Document**).

**Table 2:** Distribution of deaths by ICD-MM group of causes in SSA studies, 2015-2020

ICD-MM group and cause of death	Number of deaths	Proportion of deaths (95% confidence interval)
Group 1: Pregnancies with abortive outcome	825	7.2% (5.3%-9.1%)
Group 2: Hypertensive disorders in pregnancy	2521	22.1% (19.9%-24.2%)
Group 3: Obstetric haemorrhage	3295	28.8% (26.5%-31.2%)
Group 4: Pregnancy-related infections	1318	11.5% (9.8%-13.2%)
Group 5: Other obstetric complications	576	5.0% (3.1%-7.0%)
Group 6: Unanticipated complications of management	452	4.0% (1.6%-6.3%)
Group 7: Non-obstetric complications	2151	18.8% (16.4%-21.2%)
Group 8: Unknown/undetermined causes	289	2.5% (0.2%-4.9%)
Group 9: Coincidental causes	4	0% (0.0%-0.0%)
Total	11 431	100%

### Leading causes of maternal deaths in SSA regions

Obstetric haemorrhage was the leading cause of maternal death in each of the three SSA regions. Hypertensive disorders in pregnancy were the second leading cause of death in East and Central Africa and West Africa regions and third in Southern Africa, where non-obstetric complications were the second leading cause. Pregnancy-related infections were the fourth leading cause in all three regions (**Table 3**).

**Table 3:** Leading causes of death in studies reporting causes of maternal deaths in sub-Saharan Africa sub-regions

	Region and leading causes of death (ICD-MM groups)					
**Cause of death group**	**East and Central Africa**		**Southern Africa**		**West Africa**	
	**Proportion (95% CI)**	**Rank**	**Proportion (95% CI)**	**Rank**	**Proportion (95% CI)**	**Rank**
Obstetric haemorrhage	28.4% (19.6%-37.2%)	1	25.2% (13.1%-37.4%)	1	31.3% (26.5%-36.0%)	1
Hypertensive disorders of pregnancy	27.2% (18.5%-35.9%)	2	17.8% (7.1%-28.5%)	3	22.7% (18.4%-27.0%)	2
Non-obstetric complications	15.3% (8.3%-22.4%)	3	22.9%(9.2%-36.7%)	2	14.4% (9.5%-19.3%)	3
Pregnancy-related infections in pregnancy	11.8% (4.8%-18.9%)	4	8.8%(0.9%-16.7%)	4	13.4% (9.9%-17.0%)	4

CI – confidence interval

### Comparison of the leading causes of maternal deaths in this study, WHO and GBD studies

The leading groups of maternal death causes (obstetric haemorrhage, hypertensive disorders in pregnancy, and non-obstetric complications) were the same in our study and the WHO and the GBD (**Table 4**). The three death causes were the same in the three studies, although the top-ranking cause differed for the WHO study. The causes ranked fourth to sixth were the same for the WHO and our study. Proportions of deaths were different but 95% CIs overlapping for several groups of causes. Notably, WHO and GBD studies did not report deaths due to unanticipated management complications, unknown/undetermined, and coincidental causes.

**Table 4:** Comparison of causes of death with global studies

	Point estimates (95% confidence intervals) of proportions of deaths due to each cause, comparison of CIs and rankings for this and WHO/GDB study					
**ICD-MM Group cause of death**	**This study**		WHO study (2014)*		**GBD study (2017)†**	
	**Percent (95% CI)**	**Ranking**	**Percent (95% CI)**	**Ranking**	**Percent (95% CI)**	**Ranking**
Group 3: Obstetric haemorrhage	28.8% (26.5%-31.2%)	1	24.5% (16.9%-34.1%)	2	43.5% (34.1%-56.1%)	1
Group 2: Hypertensive disorders in pregnancy	22.1% (19.9%-24.2%)	2	16% (11.7%-21%)	3	15.6% (10.9%-21.8%)	2
Group 7: Non-obstetric complications	18.8% (16.4%-21.2%)	3	28.6% (19.9%-40.3%)	1	14.4% (10.1%-20.3%)	3
Group 4: Pregnancy-related infections	11.5% (9.8%-13.2%)	4	10.3% (5.5%-18.5%)	4	6.1% (3.9%-9.3%)	6
Group 1: Pregnancies with abortive outcome	7.2% (5.3%-9.1%)	5	9.6% (5.1%-17.2%)	5	8.6% (5.8%-12.1%)	5
Group 5: Other obstetric complications	5.0% (3.1%-7.0%)	6	9.0% (5.1%-15.7%)	6	11.9% (7.9%-17.3%)	4
Group 6: Unanticipated complications of management	4.0% (1.6%-6.3%)	7	‡		‡	
Group 8: Unknown/undetermined causes	2.5% (0.2%-4.9%)	8	‡		‡	
Group 9: Coincidental causes	0% (0.0%-0.0%)	9	‡		‡	
**Total**	**11** **431**		**1** **310** **000**		**121** **297**	

CI – confidence interval, WHO – World Health Organisation, GBD – Global Burden of Disease

*We regrouped the causes reported in this study into the ICD-MM groups.

†The point estimate and CI data were in absolute numbers. We calculated the proportions

‡No deaths were reported in these groups.

## DISCUSSION

SSA countries struggle to collect comprehensive data on maternal mortality causes. Their major handicap is the absence of well-functioning civil registration, and vital statistics systems (CRVS) which countries require to collect these data consistently [2,4,8]. SSA health systems cannot identify all maternal deaths and assign the correct causes to these deaths [54]. Cause-of-death data are often contaminated by missing and misclassified causes. Coders misunderstand coding rules, and coding errors are common [8]. Often, coders have insufficient information on events leading to the deaths, which they require to identify the correct cause of death [3,8,55].

Our study found that the leading group causes of maternal deaths in SSA in the past decade were obstetric haemorrhage, hypertensive disorders in pregnancy, non-obstetric complications, and pregnancy-related infections. The three SSA regions had similar leading group causes of death, except Southern Africa, where non-obstetric causes were the second leading cause. Postpartum haemorrhage was the leading cause of death in the obstetric haemorrhage group. Pre-eclampsia/eclampsia was the leading cause of death in the hypertensive disorders in pregnancy group, and puerperal sepsis the leading cause in the pregnancy-related infections group.

Our findings are similar to those from other studies. The top three causes of maternal death (obstetric haemorrhage, hypertensive disorders in pregnancy, and non-obstetric complications) were the same in our study and WHO and GBD studies [1,8]. Bailey and others' 2017 global systematic review of institutional maternal deaths also found that haemorrhage and hypertensive diseases were the leading causes of maternal deaths in SSA. They accounted for nearly 40% of the deaths [9]. Similarly, a review of the causes of maternal deaths in adolescents in low and middle-income countries by Neal and others’ found the leading causes to be postpartum (obstetric) haemorrhage, hypertensive disease in pregnancy, and puerperal sepsis (pregnancy-related infection) [10].

Most of the common causes of maternal mortality in SSA are due to a lack of resources to provide quality maternal health care, in addition to patient-related factors such as acceptability and affordability of maternal health services. Haemorrhage, the leading cause of death, is typically linked to a lack of resources [1,10]. It is associated with unskilled delivery assistance, delivery in ill-equipped facilities, and a shortage of essential obstetric care materials such as transfusion blood [1,56]. These challenges are endemic in SSA, where delivery at home and in unequipped primary care facilities is typical. First delay (delay in deciding to present at a health facility) and second delay (delay to move a patient to an appropriate level of care) are common because of poor health-seeking behaviours, long distances to health facilities, and lack of transport to tertiary institutions [19,39,45]. In Guinea, maternal death odds ratios were significantly elevated in cases transferred from another hospital [19]. In Ethiopia, the second delay contributed 40%, while the third delay (delay to receive adequate care when an appropriate facility is reached) contributed 22% of maternal deaths [45].

Hypertensive disorders in pregnancy, including pre-eclampsia/eclampsia and pre-existing hypertension, are common pregnancy morbidities in SSA. These conditions should be diagnosed and treated early in antenatal care (ANC), yet, most SSA countries have low ANC coverage [20]. A study by Ataguba, which used DHS data from 35 SSA countries, showed that ANC coverages (proportion of women with a live birth in the preceding 12 months who attended the WHO recommended minimum 4 ANC visits) in SSA ranged from 39% in Ethiopia in 2016 to 92% in Ghana in 2016. Out of the 35 countries, only 11 had ANC coverage above 70%, and 9 had coverages below 50% [57]. Geographical and sub-population ANC coverages are also considerably variable in SSA [58-60]. A study in Angola showed inequalities in ANC coverage favouring rich women (difference in coverage (D) between rich and poor women = 54.2%, 95% uncertainty limits (UL)  = 49.59-58.70), educated women (Difference between women with secondary education and above, and uneducated women = 43.41; UL = 39.65-47.17), the difference between lowest and highest ANC coverage region (D = 51.7; UL = 43.56 - 59.85) and residing in urban areas (D = 34.35; UL = 30.47-38.22) [60]. Variability in ANC coverage determines maternal morbidity and mortality patterns such that sub-populations with low ANC coverage have higher maternal morbidity and mortality. Obstetric complications are not identified and treated early among women who do not attend ANC. Carroli and others’ synthesis of evidence described the effectiveness of ANC in preventing maternal mortality and severe morbidity [61]. Interventions such as prevention, detection and treatment of anaemia, detection and treatment of hypertensive disease, testing and treatment of infections (Malaria, HIV, Syphilis, other sexually transmitted infections etc.) is done in ANC to improve maternal and child health outcomes [61].

Puerperal sepsis, a bacterial infection of the genital tract from the onset of rupture of membranes or labour to 42 days post-delivery, is another important cause of death in SSA. Puerperal sepsis is common in developing countries because of the lack of basics, such as clean water, clean delivery rooms, and limited access to antibiotics. Ngonzi and others, in their study at Mbarara Regional Referral Hospital in Uganda, found puerperal sepsis as the leading cause of maternal deaths because of the erratic availability of antibiotics [38]. Even caesarean deliveries become sources of puerperal infections in SSA, as found in one of Zimbabwe's leading tertiary hospitals, where puerperal sepsis was the leading cause of death and emergency caesarean delivery was among the main factors associated with it [20]. Moodley and others in South Africa also reported caesarean delivery as an underlying factor in puerperal sepsis deaths [62].

Although the proportion of deaths due to non-obstetric complications in this study was comparable to other studies, we believe that there were numerous challenges in reporting deaths in this group. The majority of deaths in the group were reported as “other indirect causes” and unspecified. Similarly, WHO analysis also found indirect deaths to be not well delineated [8]. HIV, a significant problem in SSA, had fewer deaths than anaemia and non-pregnancy-related infections. It was unclear how the studies assigned HIV as a cause of death. A systematic review of pregnancy-related deaths attributed to HIV in SSA by Grollman and others similarly found that only four out of twelve studies that assigned HIV as a cause of death stated the method used [12]. In the WHO analysis, Say and others noted that it is often difficult to distinguish between indirect HIV deaths and direct deaths of HIV-infected women [8]. Pregnancy worsens some non-obstetric conditions like pneumonia, which is often associated with HIV. Thus, the relationship between HIV and other conditions is difficult to extricate in coding death causes [62].

Most SSA countries may be unable to report postpartum deaths widely. South Africa found many unrecorded pneumonia and Tuberculosis (TB) puerperium deaths in their demographic health surveys; among women who delivered in hospitals, got discharged and died at home [41]. SSA may also be under-reporting abortion-related deaths in ICD-MM group one. We found a considerable variation in the proportion of deaths attributed to abortion in the studies reviewed. Each of the SSA sub-regions had one study that reported high proportions of abortion-related deaths – South Africa in Southern Africa [41], Tanzania in East and Central Africa [18], and Cameroon in West Africa [32]. In the WHO analysis, Say and others noted that abortion-related deaths had a high risk of misclassification [7]. Kassebaum and others in the GBD study similarly acknowledged the challenge of quantifying abortion-related deaths [1]. Illegality and religious blacklisting of abortion in some countries makes it underreported or misclassified [7].

Our review found only four deaths of incidental causes and about 3% of the unknown/undetermined causes. While deaths of incidental causes do not contribute to MMR estimates and cause-of-death analysis, it is essential to measure these deaths accurately. Studies that misclassify incidental deaths as maternal inflate MMR estimates and studies that misclassify maternal deaths as incidental deflate MMR estimates. As a result, WHO includes deaths of unknown/undetermined causes in MMR estimates but excludes them from cause-of-death analysis [2]. Misclassification of deaths into unknown/undetermined cause group removes the deaths from their correct causes. Consequently, deaths are under-counted in the specific cause groups.

Studies in our review may have reported fewer deaths in the unknown/undetermined cause group because they mainly analysed institutional deaths. Even so, a few studies reported 30% to 45% deaths in the unknown/undetermined group [50,63]. Community studies such as demographic and health surveys typically report deaths in the unknown/undetermined group because they cannot identify death causes. Scanty information also leads to lumping deaths in the unknown/undetermined group [8,64]. Most institutional deaths occur at tertiary facilities where the women are referred from lower-level facilities, often with inadequate records [39]. The records are often missing or incompletely or inaccurately documented, without vital indicators and details of events leading to death [1,2,4]. When audit committees assess the death causes, they need such information. The capacity to diagnose and assign correct death causes is also weak in SSA countries [65,66]. Specialist clinicians with higher competency to diagnose causes of death are in few tertiary hospitals. As a result, deaths occurring at primary care levels often receive incompetent cause-of-death diagnoses.

Our study may have some limitations. It identified and quantified the causes of maternal deaths presented in published literature, where inadequate publishing of data on causes of maternal deaths in some countries was a potential limitation. In addition, all quantified cause-of-death analyses are to be used with caution because it is difficult to produce precise estimates. Scantiness and poor data quality compromises the estimates [3,8,55]. WHO and GBD reviews did not report deaths in the unanticipated management complications, unknown/undetermined, and coincidental causes groups because of these challenges. The strength of our review is that it identified a considerable number of studies from all SSA regions, which made the study produce results comparable to those from WHO and GBD studies. It also allowed a comparison of the leading causes in the SSA regions [67].

Our findings have important implications for policy, practice and future research. The study has identified the leading causes of maternal deaths in SSA, which governments and development partners are to prioritise in maternal mortality interventions. Analysis of the causes of maternal deaths using data from published studies has exposed a critical gap in the availability of these data, which has implications for future research and documentation. The findings of this review showed the need for increased publication of data on causes of maternal deaths in SSA. While many SSA governments are engaged in CEMD and MDSR initiatives, the data are not easily accessible. This has to improve. A central repository of government reports and publicly available data sets would be helpful in future reviews of this kind.

## CONCLUSION

Obstetric haemorrhage, hypertensive disorders in pregnancy, non-obstetric complications, and pregnancy-related infections are the leading causes of maternal death in SSA. Postpartum haemorrhage is the leading cause of death in the obstetric haemorrhage group. Pre-eclampsia/eclampsia is the leading cause in the hypertensive disorders in pregnancy group, and puerperal sepsis the leading cause in the pregnancy-related infections group. Challenges exist in reporting deaths due to abortion-related complications, non-obstetric complications and incidental causes. SSA must invest in building health information systems that collect and avail comprehensive and quality data on all causes of maternal deaths.

## Additional material


Online Supplementary Document
