# Supplementary material for: Causes of maternal mortality in Sub-Saharan Africa: A systematic review of studies published from 2015 to 2020
Source: J Glob Health. 2021 Oct 9;11:04048. doi: 10.7189/jogh.11.04048 (PMC8542378; doi:10.7189/jogh.11.04048)
Supplement: Online Supplementary Document [file jogh-11-04048-s001.pdf]

Online Supplementary Document

Table S1: Detailed search strategy for each search.

PubMed search by MeSH terms for articles from Africa (last search date: 21/05/2021)	((((((reproductive age[MeSH Terms]) OR (pregnancy-related[MeSH Terms])) OR (maternal[MeSH Terms])) AND (death[MeSH Terms])) OR (mortality[MeSH Terms])) AND (causes[MeSH Terms])) AND (Africa[MeSH Terms])) AND (("2015/01/01"[Date - Publication] : "2020/12/31"[Date - Publication]))
PubMed search by MeSH terms for articles by SSA country names, one by one (last search date: 21/05/2021)	((((((reproductive age[MeSH Terms]) OR (pregnancy-related[MeSH Terms])) OR (maternal[MeSH Terms])) AND (death[MeSH Terms])) OR (mortality[MeSH Terms])) AND (causes[MeSH Terms])) AND (Sub-Saharan Africa country name[MeSH Terms])) AND (("2015/01/01"[Date - Publication] : "2020/12/31"[Date - Publication]))
PubMed search by MeSH terms for articles for all SSA country names (last search date: 21/05/2021)	((((((((reproductive[MeSH Terms]) OR (pregnancy[MeSH Terms])) OR (maternal[MeSH Terms])) AND (mortality[MeSH Terms])) OR (death[MeSH Terms])) AND (causes[MeSH Terms])) OR (aetiology[MeSH Terms])) AND ("Angola"[Text Word] OR "Botswana"[Text Word] OR "Benin"[Text Word] OR "Burkina Faso"[Text Word] OR "Burundi"[Text Word] OR "Cameroon"[Text Word] OR "Cape Verde"[Text Word] OR "Central African Republic"[Text Word] OR "Chad"[Text Word] OR "Comoros"[Text Word] OR "Cote d'Ivoire"[Text Word] OR "Congo"[Text Word] OR "Congo Brazzaville"[Text Word] OR "Democratic Republic of the Congo"[Text Word] OR "Djibouti"[Text Word] OR "Equatorial Guinea"[Text Word] OR "Eritrea"[Text Word] OR "Ethiopia"[Text Word] OR "Gabon"[Text Word] OR "Ghana"[Text Word] OR "Guinea Bissau"[Text Word] OR "Guinea Conakry"[Text Word] OR "Kenya"[Text Word] OR "Lesotho"[Text Word] OR "Liberia"[Text Word] OR "Madagascar"[Text Word] OR "Malawi"[Text Word] OR "Mali"[Text Word] OR "Mauritania"[Text Word] OR "Mauritius"[Text Word] OR "Mozambique"[Text Word] OR "Namibia"[Text Word] OR "Niger"[Text Word] OR "Nigeria"[Text Word] OR "Reunion"[Text Word] OR "Rwanda"[Text Word] OR "Sao Tome and Principe"[Text Word] OR "Senegal"[Text Word] OR "Seychelles"[Text Word] OR "Sierra Leone"[Text Word] OR "Somalia"[Text Word] OR "South Africa"[Text Word] OR "South Sudan"[Text Word] OR "Sudan"[Text Word] OR "Swaziland"[Text Word] OR "Eswatini"[Text Word] OR "Tanzania"[Text Word] OR "The Gambia"[Text Word] OR "Gambia"[Text Word] OR "Togo"[Text Word] OR "Uganda"[Text Word] OR "Western Sahara"[Text Word] OR "Zambia"[Text Word] OR "Zimbabwe"[Text Word])) AND (("2015/01/01"[Date - Publication] : "2020/12/31"[Date - Publication]))
World Cat Discovery search (last search date: 21/05/2021)	kw:(reproductive) OR kw:(pregnancy) OR kw:(maternal) AND kw:(mortality) OR kw:(death) AND kw:(causes) OR kw:(etiology) AND kw:(Africa) AND (yr:2015..2020).<br>Filters: English language, peer-reviewed articles, all databases, include related terms.

Google Scholar search (last search date: 21/05/2021)	"reproductive age" or "pregnancy related" or "maternal" and "mortality" or "death" and "causes" or "etiology" and "Africa". Filters: time custom range 2015-2020.

Table S2: Data extraction form for systematic review of maternal mortality causes in SSA

**Study details**													
													**1 V**
**Author, Year published**	**Study description**	**Study implementer**	**Data period**	**Study design**	**Study setting**	**MDs data source**	**MD definition**	**Community deaths included**	**Method of assigning cause of death**	**Country**	**Region**	**Percent complete cause of death data**	**Un Se co ati abo**
Vousden, 2020	A secondary analysis of a stepped-wedge cluster randomised controlled trial	Study team	April 2016 to November 2017	stepped-wedge cluster randomised controlled trial	secondary or tertiary health facility and peripheral referral facilities	Health facility records and verbal autopsy	ICD-10 MM	Yes	Expert panel	Ethiopia, Uganda, Malawi, Sierra Leone, Zambia, Zimbabwe	Multi-country	100%	
Samuels, 2020	A study to determine the frequency of maternal near-miss (MNM) and maternal deaths	Study team	June 2012 to May 2013	retrospective records review	Jos University Teaching Hospital	Health facility records	ICD-10 MM	No	Not stated	Nigeria	West Africa	100%	
Said, 2020	A cross-sectional study of MDSR reviewed deaths	Study team	Jan - Dec 2018	MDSR audits review	Lindi and Mtwara regions (Referral, district, mission hospitals	MDSR audits and verbal autopsy	ICD-10 MM	Yes	Expert panel	Tanzania	East Africa	100%	

					and health centres)							
Nassoro, 2020	A retrospective review of records in a regional referral hospital	Study team	Jan-Dec 2018	retrospective records review	Regional Referral Hospital	Health facility records	Not stated	No	Hospital MDSR committee	Tanzania	East Africa	100%
Hadush, 2020	A national survey of emergency obstetric and new-born care	Public institute	May 2016 - Dec 2016	cross-sectional facility study	Health facilities in 9 regions	MDSR audits	Not stated	No	Expert panel	Ethiopia	East Africa	100%
Gebretsadik, 2020	Evaluating the causes and contributors to maternal death at a specialised hospital	Study team	Jan 2016 - Aug 2017	retrospective records review	Hawassa Comprehensive Specialised Hospital,	Health facility records	ICD-10 MM	No	Expert panel	Ethiopia	East Africa	100%
RSA NCCEMD, 2017	Vital registration and NCCEMD data	Government department	2015-16	CEMD and VR	National	CEMD audits	ICD-10 MM	Yes	CEMD	South Africa	Southern Africa	100%
Zim MDSR, 2019	MPDSR national reports, 2016-2018; 2019	Government department	2016-2019	MDSR audits review	National	MDSR audits	ICD-10 MM	Yes	MDSR audits	Zimbabwe	Southern Africa	81%
Bwana, 2019	Maternal mortality causes and trends in public hospitals	Study team	2011-2015	retrospective records review	34 hospitals -national, zonal and regional	Health facility records	ICD-10 MM	No	Expert panel	Tanzania	East Africa	100%
Diallo, 2020	Maternal mortality risk indicators: Case-control study at a referral hospital in Guinea	Study team	Jan 2016 - Oct 2017	retrospective records review	Gnace Deen National Hospital in Conakry	Health facility records	Not stated	No	Facility clinical assessments	Guinea	West Africa	99%

Ngwenya, 2020	Factors associated with maternal mortality from sepsis: a five-year review	Study team	Jan 2012-Dec 2016	retrospective records review	Mpilo Central Hospital, Bulawayo	Health facility records	Not stated	No	Facility clinical assessments	Zimbabwe	Southern Africa	100%	
Uchendu, 2020	Mortality synopsis Among women of reproductive age group	Study team	Jan 2016 - Dec 2018	retrospective records review	Delta State university Teaching Hospital	Health facility records	ICD-10 MM	No	Expert panel	Nigeria	West Africa	100%	
Uzabakiriho, 2019	Causes of maternal deaths at Natalspruit Hospital,	Study team	Jan 2013 to Dec 2014	retrospective records review	Natalspruit Hospital, Johannesburg,	Health facility records	ICD-10 MM	No	Expert panel	South Africa	Southern Africa	100%	
Sageer, 2019	Causes and contributory factors of maternal mortality after state-wide MPDSR review	Study team	Jan 2015 to Dec 2016	retrospective records review	Ogun State in South western Nigeria	MDSR audits	ICD-10 MM	No	MDSR committees	Nigeria	West Africa	100%	
Oyeneyin, 2019	Maternal mortality ratios, causes of deaths, geographical distribution and associated factors in 12-month CEMD reports	Study team	June 2012 to May 2015	retrospective records review	Ondo State	Confidential Enquiry into Maternal Deaths(CEMD) audits	ICD-10 MM	No	Expert panel	Nigeria	West Africa	100%	
Nkhwalume, 2019	Trends of MMR at Princess Marina and Nyangabwe referral hospitals, before	Study team	Sept 2010 - Dec 2011; Sept 2012 - Dec 2013	Pre and Post evaluation	two national referral hospitals in Gaborone and Nyangabwe	Health facility records	Not stated	No	Facility audit teams	Botswana	Southern Africa	100%	

	and after EMOC training												
Aikpitanyi, 2019	Medical causes and contributory factors of maternal mortality from MPDSR at the Central Hospital	Study team	Oct 2017 to May 2019	retrospective records review	Benin city central hospital	Health facility records	Not stated	No	Facility MPDSR committee	Nigeria	West Africa	100%	
Aden, 2019	Causes and contributing factors of maternal deaths	Study team	Jan-Dec 2016	retrospective records review and verbal autopsy	Bosaso District, Puntland State	Health facility records	ICD-10 MM	No	Expert panel	Somalia	East Africa	100%	
Zamane, 2018	Causes of maternal deaths in the Dori Regional Hospital	Study team	Jan 2014 to Dec 2016	retrospective records review	Dori hospital, Regional Hospital	Health facility records	Not stated	No	Facility clinical assessments	Burkina Faso	West Africa	100%	
Imran, 2018	Population-based rates, timing, and causes of maternal deaths, stillbirths, and neonatal deaths in south Asia and sub-Saharan Africa (SSA)	Study team	July 2012 to Feb 2016	Prospective cohort study	6 SSA countries	Verbal autopsy	ICD-10 MM	Yes	Expert panel	SSA (DRC, Ghana, Kenya, Tanzania, and Zambia)	Multi-country	100%	
Awowole, 2018	Maternal mortality analysis at a university teaching hospital	Study team	Oct 2012 to Sept 2015	retrospective records review	Obafemi Awolowo University Hospitals	Health facility records	ICD-10 MM	No	Expert panel	Nigeria	West Africa	100%	
Agan, 2018	Trends and causes of maternal	Study team	2010 to 2014	retrospective records review	University of Calabar Teaching	Health facility records	ICD-10 MM	No	Expert panel	Nigeria	West Africa	100%	

	mortality in a tertiary hospital: A 5-year retrospective study				Hospital, Calabar, Nigeria								
Ntoimo, 2018	Prevalence and risk factors for maternal mortality in referral hospitals in eight states	Study team	Jan – Jun 2014	retrospective records review	national multi-center study conducted in eight referral hospitals	Health facility records	ICD-10 MM	No	Facility clinical assessments	Nigeria	West Africa	99%	
Okonofua, 2017	Maternal death review and outcomes assessment in Lagos	Study team	Jan 2015 - Sept 2016	retrospective records review	three public maternity hospitals - AGH, GGH and LIMH	MDSR audits	ICD-10 MM	No	MPDSR committees	Nigeria	West Africa	100%	
Kamga, 2017	Role of abortion and ectopic pregnancies in maternal mortality rate at	Study team	Jun 2011 to May 2016	retrospective records review	Three hospitals in the city of Yaoundé: HCY, CHU, HGOPY.	Health facility records	Not stated	No	Facility clinical assessments	Cameroon	West Africa	100%	
Idoko, 2017	A retrospective analysis of trends in maternal mortality in tertiary health centre	Study team	Jan 2011 - Dec 2014	retrospective records review	Edward Francis Small Teaching Hospital	Health facility records	WHO ICD MM	No	Expert panel	Gambia	West Africa	96%	
Owusu-Sarpong, 2017	Associated factors and quality of care among maternal deaths at a regional hospital	Study team	Jan-Dec 2012	retrospective records review	The Eastern Regional hospital	Maternal death audits	Not stated	No	Expert panel	Ghana	West Africa	100%	

Orazulike, 2017	Study of mortality in women of reproductive age in a tertiary health facility	Study team	Jan 2013-Dec 2015	retrospective records review	University of Port Harcourt Teaching Hospital	Health facility records	ICD-10 MM	No	Expert panel	Nigeria	West Africa	100%	
Umar, 2016	Maternal Mortality in the main referral hospital	Study team	2010 to 2014	retrospective records review	Kuando Kubango province	Health facility records	ICD-10 MM	No	Not stated	Angola	Southern Africa	76%	
Ngonzi, 2016	Analysis of the leading cause of maternal deaths at a tertiary university Teaching Hospital	Study team	Jan 2011 - Nov 2014	case-control study	Mbarara Regional Referral Hospital (MRRH), Sothern Uganda	Health facility records	ICD-10 MM	No	Not stated	Uganda	East Africa	99%	
Sayinzoga, 2016	Nationwide facility-based retrospective cohort study of maternal deaths	Govt department	Jan 2010 - Dec 2013	MDSR audits	national study	MDSR audits	ICD-10 MM	No	District audit committees	Rwanda	East Africa	100%	
Mgawadere, 2016	Measuring maternal mortality using a Reproductive Age Mortality Study (RAMOS)	Study team	Dec 2011 to Nov 2012	RAMOS facility and community study	Mangochi District	Health facility records and verbal autopsy	ICD-10 MM	Yes	Expert panel	Malawi	Southern Africa	100%	
Adomako, 2016	Community-based surveillance of maternal deaths in rural Ghana	Study team	May and Aug 2013	RAMOS facility / community study	Bosomtwe district	Vebal autopsy	ICD-10 MM	Yes	Expert panel	Ghana	West Africa	100%	
Oladapo, 2015	Nationwide cross-sectional study of maternal deaths	Study team	Jun 2012 to Aug 2013	cross-sectional facility study	46 public tertiary hospitals in the six geopolitic	Health facility records	ICD-10 MM	No	Expert panel	Nigeria	West Africa	100%	

	and near-misses in public tertiary hospitals				al zones of Nigeria								
Adeniran, 2015	Predictors of maternal mortality among critically ill obstetric patients	Study team	Jan 2010 - Jun 2013	retrospective records review	University of Ilorin Teaching Hospital, Ilorin	Health facility records	Not stated	No	Expert panel	Nigeria	West Africa	100%	
Rulisa, 2015	Prevalence and factors associated with severe maternal near-miss morbidity and mortality study	Study team	Oct 2010-Oct 2011	cross-sectional facility study		Health facility records and verbal autopsy	Not stated	No	Expert panel	Rwanda	East Africa	100%	
Godefay, 2015	Analysis of deaths among women aged 15-49 years retrospectively over a one-year period	Study team	May 2012 and April 2013, collected mid-2013	cross-sectional community survey	Tigray Region, in northern Ethiopia	Verbal autopsy	Not stated	Yes	Expert panel	Ethiopia	East Africa	84%	
Bello, 2015	Maternal mortality audit in a Tertiary Health Institution	Study team	2012 - 2014	prospective facility study	tertiary referral hospital in South-West Nigeria,	Health facility records	ICD-10 MM	No	Expert panel	Nigeria	West Africa	100%	
**Total**													

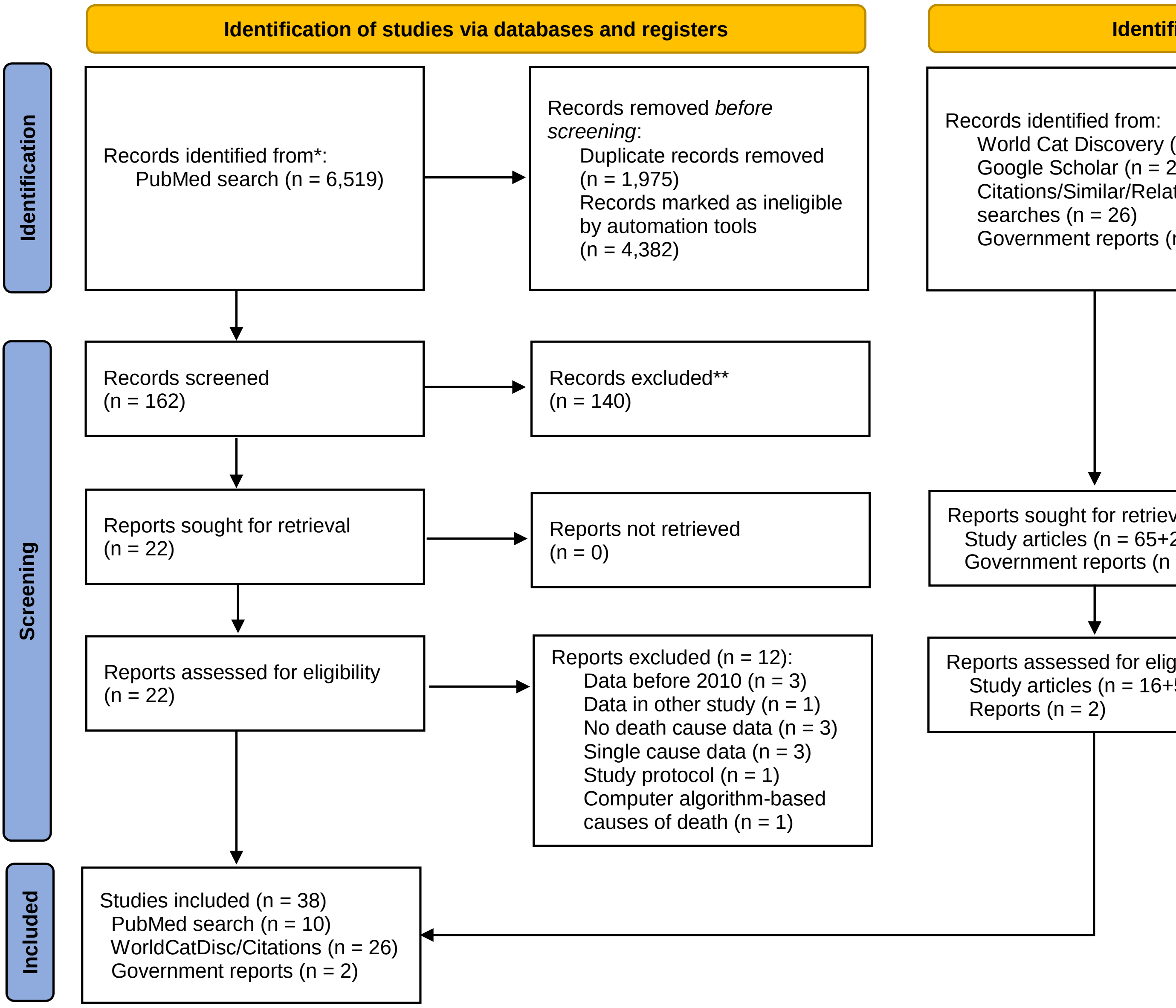
Identification of studies via databases and registers
Identifi
Identification
Records identified from*:
PubMed search (n = 6,519)
Records removed *before screening*:
Duplicate records removed (n = 1,975)
Records marked as ineligible by automation tools (n = 4,382)
Records identified from:
World Cat Discovery (
Google Scholar (n = 2
Citations/Similar/Relat
searches (n = 26)
Government reports (r
Screening
Records screened (n = 162)
Records excluded** (n = 140)
Reports sought for retrieval (n = 22)
Reports not retrieved (n = 0)
Reports sought for retriev
Study articles (n = 65+2
Government reports (n
Reports assessed for eligibility (n = 22)
Reports excluded (n = 12):
Data before 2010 (n = 3)
Data in other study (n = 1)
No death cause data (n = 3)
Single cause data (n = 3)
Study protocol (n = 1)
Computer algorithm-based causes of death (n = 1)
Reports assessed for elig
Study articles (n = 16+
Reports (n = 2)
Included
Studies included (n = 38)
PubMed search (n = 10)
WorldCatDisc/Citations (n = 26)
Government reports (n = 2)

Figure 1.

Table S3: Risk of bias assessment in the studies reporting maternal mortality causes in sub-Saharan Africa, 2015-2020.

**Study**	**Risk of bias and rating for each criteria (risk rating)**				
	**Source of data (information bias)**	**Completeness of cause-of-death data (missing data bias)**	**Method of assigning causes of death (selection bias)**	**Competence in assigning the causes of death (measurement bias)**	**Overall (avera**
Vousden, 2020.	Health facility records and verbal autopsy (1)	100% (1)	ICD-MM (1)	Study expert panel (1)	
Samuels, 2020	Health facility records (1)	100% (1)	ICD-MM (1)	Not stated (3)	M
Said, 2020	MDSR audits and verbal autopsy (2)	100% (1)	ICD-MM (1)	Study expert panel (1)	(
Nassoro, 2020	Health facility records (1)	100% (1)	Not stated (2)	Hospital MDSR committee (1)	(
Hadush, 2020	MDSR audits (2)	100% (1)	Not stated (2)	Study expert panel (1)	M
Gebretsadik, 2020	Health facility records (1)	100% (1)	ICD-MM (1)	Study team (1)	
RSA, NCCEMD, 2017	CEMD audits (2)	100% (1)	ICD-MM (1)	CEMD (1)	(
Zim MDSR, 2019	MDSR audits (2)	81% (2)	ICD-MM (1)	MDSR audits (1)	M
Bwana, 2019	Health facility records (1)	100% (1)	ICD-MM (1)	Study expert panel (1)	
Diallo, 2020	Health facility records (1)	99% (1)	Not stated (2)	Facility clinical assessments (2)	M
Ngwenya, 2020	Health facility records (1)	100% (1)	Not stated (3)	Facility clinical assessments (2)	M (
Uchendu, 2020	Health facility records (1)	100% (1)	ICD-MM (1)	Study expert panel (1)	
Uzabakiriho, 2019	Health facility records (1)	100% (1)	ICD-MM (1)	Study expert panel (1)	
Sageer, 2019	MDSR audits (2)	100% (1)	ICD-MM (1)	MDSR committees (1)	(

Study	Risk of bias and rating for each criteria (risk rating)				
	Source of data (information bias)	Completeness of cause-of-death data (missing data bias)	Method of assigning causes of death (selection bias)	Competence in assigning the causes of death (measurement bias)	Overall (avera[illegible]
Oyeneyin, 2019	CEMD audits (2)	100% (1)	ICD-MM (1)	Study expert panel (1)	([illegible]
Nkhwalume, 2019	Health facility records (1)	100% (1)	Not stated (3)	Facility audit teams (1)	M[illegible]
Aikpitanyi, 2019	Health facility records (1)	100% (1)	Not stated (2)	Facility audit teams (1)	([illegible]
Aden, 2019	Health facility records (1)	100% (1)	ICD-MM (1)	Study expert panel (1)	[illegible]
Zamane, 2018	Health facility records (1)	100% (1)	Not stated (2)	Facility clinical assessments (2)	M[illegible]
Imran, 2018	Verbal autopsy (3)	100% (1)	ICD-MM (1)	Physician verbal autopsy coders (1)	M[illegible]
Awowole, 2018	Health facility records (1)	100% (1)	ICD-MM (1)	Study expert panel (1)	[illegible]
Agan, 2018	Health facility records (1)	100% (1)	ICD-MM (1)	Study expert panel (1)	[illegible]
Ntoimo, 2018	Health facility records (1)	99% (1)	ICD-MM (1)	Facility clinical assessments (2)	([illegible]
Okonofua, 2017	MDSR audits (2)	100% (1)	ICD-MM (1)	MPDSR committees (1)	M[illegible] ([illegible]
Kamga, 2017	Health facility records (1)	100% (1)	Not stated (3)	Facility clinical assessments (1)	M[illegible]
Idoko, 2017	Health facility records (1)	96% (1)	WHO ICD MM (1)	Study expert panel (1)	[illegible]
Owusu-Sarpong, 2017	Maternal death audits (1)	100% (1)	Not stated (3)	Study expert panel (1)	M[illegible]
Orazulike, 2017	Health facility records (1)	100% (1)	ICD-MM (1)	Study expert panel (1)	[illegible]
Umar, 2016	Health facility records (1)	76% (1)	ICD-MM (1)	Not stated (3)	M[illegible]
Ngonzi, 2016	Health facility records (1)	99% (1)	ICD-MM (1)	Not stated (3)	M[illegible]

Study	Risk of bias and rating for each criteria (risk rating)				
	**Source of data (information bias)**	**Completeness of cause-of-death data (missing data bias)**	**Method of assigning causes of death (selection bias)**	**Competence in assigning the causes of death (measurement bias)**	**Overall (avera**
Sayinzoga, 2016	MDSR audits (2)	100% (1)	ICD-MM (1)	District audit committee (1)	(
Mgawadere Florence, 2016	Health facility records and verbal autopsy (1)	100% (1)	ICD-MM (1)	Study expert panel (1)	
Adomako, 2016	Verbal autopsy (3)	100% (1)	ICD-MM (1)	Study expert panel (1)	M
Oladapo, 2015	Health facility records (1)	100% (1)	ICD-MM (1)	Study expert panel (1)	

Table S4: Number of studies reporting causes of maternal mortality in sub-Saharan Africa, by country of study, 2015-2020.

**Country**	**Number of studies**	**Number of maternal deaths**	**Proportion of deaths**	**Country**	**Number of studies**	**Number of maternal deaths**	**Proportion of deaths**
Angola	1	99	1%	Multi-country*	1	940	8%
Botswana	1	74	1%	Multi-country†	1	213	2%
Burkina Faso	1	141	1%	Nigeria	13	1996	18%
Cameroon	1	414	4%	Rwanda	2	863	8%
Ethiopia	3	720	6%	Somalia	1	30	0%
Gambia	1	316	3%	South Africa	2	2405	21%
Ghana	2	101	1%	Tanzania	3	1727	15%
Guinea	1	126	1%	Uganda	1	138	1%
Malawi	1	86	1%	Zimbabwe	2	1072	9%
				**Grand Total**	**38**	**11431**	**100%**

* Ethiopia, Uganda, Malawi, Sierra Leone, Zambia, Zimbabwe

† Democratic Republic of Congo, Ghana, Kenya, Tanzania, Zambia

Table S5: Distribution of deaths by specific cause in studies reporting causes of maternal deaths in sub-Saharan Africa, 2015-2020.

Cause of death*	Number	Proportion of deaths	Lower limit	Upper limit
**Group 1: Pregnancies with abortive outcome**				
Unsafe/septic /complications of abortion	643	0.06	0.04	0.07
Ectopic Pregnancy	182	0.02	0.00	0.03
**Group 2: Hypertensive disorders in pregnancy**				
hypertensive disease in pregnancy	1238	0.11	0.09	0.13
Severe/Pre-eclampsia/Eclampsia	1283	0.11	0.08	0.14
**Group 3: Obstetric haemorrhage**				
Obstetric haemorrhage	1282	0.11	0.08	0.14
Postpartum haemorrhage/PPH	1533	0.13	0.11	0.16
Antepartum haemorrhage/APH	156	0.01	0.00	0.04
Ruptured uterus	323	0.03	0.01	0.05
**Group 4: Pregnancy-related infections**				
Pregnancy-related infections	227	0.02	0.00	0.06
Puerperal Sepsis	1091	0.10	0.08	0.11
**Group 5: Other obstetric complications**				
Obstructed/prolonged labor	207	0.02	0.00	0.04
Obstetric embolism	150	0.01	0.00	0.04
Peripartum cardiomyopathy	25	0.00	0.00	0.02
Other direct causes	194	0.02	0.00	0.04
**Group 6: Unanticipated complications of management**				
Anesthetic complications/High spinal anesthesia	113	0.01	0.00	0.03
Medical and surgical disorders	339	0.03	0.00	0.07
Anaemia/severe anaemia	395	0.03	0.02	0.05
**Group 7: Non-obstetric complications**				
Heart disease/Cardiac disease	88	0.01	0.00	0.03
HIV/AIDS	164	0.01	0.00	0.03
Malaria	144	0.01	0.00	0.03
Pneumonia/Jirovecii pneumonia	46	0.00	0.00	0.02
Non-pregnancy related infections	635	0.06	0.00	0.21
Other indirect causes	679	0.06	0.04	0.08
**Group 8: Unknown/undetermined causes**				
Unknown/unspecified causes	289	0.025	0.02	0.05
**Group 9: Coincidental causes**				
Incidental	4	0.00	0.00	0.02

*Some of the causes are stated in the alternative ways that they were given in the studies
